# Expectations of children and adolescents suffering from cancer

**DOI:** 10.1192/j.eurpsy.2024.915

**Published:** 2024-08-27

**Authors:** G. Kamperopoulou, A. Zartaloudi, M. Moschovi, C. Tzoumaka-Bakoula, E. Dousis, E. Evangelou, C. Dafogianni, M. Polikandrioti, I. Koutelekos

**Affiliations:** ^1^ Greek Health System; ^2^Department of Nursing, University of West Attica; ^3^Department of Medicine, National and Kapodistrian University of Athens, Athens, Greece

## Abstract

**Introduction:**

Investigation expectations for children and adolescents with cancer is an important issue for their psycho-emotional development as well as their quality of life.

**Objectives:**

To investigate the expectations of children suffering from cancer.

**Methods:**

102 questionnaires were collected from pediatric patients suffering from neoplasia disease (62 boys and 40 girls) with a median age of 13 years, covering the multidimensional expectation questionnaire (MEQ) suitable for children with cancer in a 4-point Likert scale. The MEQ was then evaluated using the SPSS.21 statistical package, which resulted in 13 questions. The questionnaire of expectations highlighted three factors that referred to the “family life expectations”, “daily life / daily routine and career prospects”, and “expectations of networking friendship”, respectively. The statistical results were obtained by multi-line regression analysis, with the Stata 12.1 statistical package, while ethical issues were complied with and licensed.

**Results:**

MEQ reliability (Cronbach’s alpha) for the entire scale was 0.82 and for agents ranged from 0.65-0.84. Overall, pediatric cancer patients delivered a fairly high average score of 3,33 ± 0,42 questions in the expectation’s questionnaire, while the mean scores were 3,29 ± 0,63, 3,51 ± 0, 45 and 3.19 ± 0.54, respectively. From the results of the analysis of multiple regression, it appeared that, as the age increases, the patients with neoplastic disease have overall 76 lower expectations (p = 0.014), while the satisfaction of the doctors-nursing staff in the total expectations is positive (p = 0.018). In the family life expectancy factor, the age of children appears to play a negative role in increasing age (p = 0.019), while positive body image and satisfaction with doctors-nursing staff (p = 0.040, p = 0.006) respectively. It appeared that children aged> 13 years have worse outcomes in expectations of the daily routine and career prospects with (p = 0.037).

**Conclusions:**

The MEQ has proven to be a valid and reliable tool that can provide pediatric staff and researchers with information about the expectations of children and adolescents with cancer that require long-term health care.

**Disclosure of Interest:**

None Declared

